# LncRNA TTN-AS1 promotes migration, invasion, and epithelial mesenchymal transition of lung adenocarcinoma via sponging miR-142-5p to regulate CDK5

**DOI:** 10.1038/s41419-019-1811-y

**Published:** 2019-07-30

**Authors:** Yunlong Jia, Yuqing Duan, Tianxu Liu, Xuexiao Wang, Wei Lv, Mengjie Wang, Jiali Wang, Lihua Liu

**Affiliations:** Department of Tumor Immunotherapy, Fourth Hospital of Hebei Medical University and Hebei Cancer Institute, Shijiazhuang, 050035 Hebei China

**Keywords:** Non-small-cell lung cancer, Oncogenes

## Abstract

Emerging evidence suggests that long noncoding RNA (lncRNA) plays pivotal roles in regulating various biological process in human cancers. Titin-antisense RNA1 (TTN-AS1) has been regarded as a tumor promoting lncRNA in numerous cancers. However, the clinical significance and biological function of TTN-AS1 in lung adenocarcinoma (LUAD) remain unclear. In the present study, we revealed that the expression of TTN-AS1 was upregulated in LUAD tissues and cell lines. High TTN-AS1 expression was associated with TNM stage and lymph node metastasis of LUAD patients. In addition, high expression of TTN-AS1 was correlated with poor postoperative prognosis of LUAD patients. Knockdown of TTN-AS1 significantly inhibited the growth, proliferation, migration, and invasion ability of LUAD cells in vitro. Then, by using bioinformation analysis and luciferase reporter experiment, we identified that TTN-AS1 could function as a competing endogenous RNA (ceRNA) by sponging miR-142-5p to regulate the expression of cyclin-dependent kinase 5 (CDK5) in LUAD. Since CDK5 is a key regulator in the process of epithelial mesenchymal transition (EMT), we detected the expression of EMT-related proteins, consequently, EMT was suppressed by knockdown of TTN-AS1 while this phenomenon was rescued by miR-142-5p inhibitor. Taken above, our study revealed that TTN-AS1 played an important role in LUAD progression. TTN-AS1/miR-142-5p/CDK5 regulatory axis may serve as a novel therapeutic target in the treatment of LUAD.

## Introduction

Lung cancer is the most common malignant tumor, constituting the leading cause of cancer-related death worldwide. Non-small cell lung cancer (NSCLC) accounts for 85% of lung cancers, and adenocarcinoma is the predominant histologic subtype at present^1^. Although great progress has been made in diagnosis and treatment recently, the long-term clinical prognosis and overall survival of lung adenocarcinoma (LUAD) patients remain unsatisfactory^2^. The inadequate comprehension of LUAD-related biological mechanisms limits the improvement in therapeutic efficacies. Thus, it is urgent to further elucidate the involved mechanisms of cancer progression to improve the clinical outcome of LUAD patients.

Aberrant expressions of oncogene and tumor suppressor gene are frequently observed in NSCLC and account for the carcinogenesis^2,3^. Benefiting from the advances in high throughput analysis, increasing cancer-related long non-coding RNAs (lncRNAs) have been identified^4^. LncRNAs are defined as a class of transcripts longer than 200 nucleotides that lack any detectable open reading frame^5^. Accumulative studies have proved that lncRNAs exert pivotal roles in the regulation of gene expression. Aberrant expression of various lncRNAs have been identified in different tumors, including colorectal cancer, gastric cancer, breast cancer, NSCLC, etc.^6–9^ lncRNAs could function as either oncogene or tumor suppressor gene to participate in the pathogenesis and development of tumors, and some lncRNAs might be potential biomarkers for predicting tumor invasion and metastasis^10,11^. Certain differentially expressed lncRNAs may function as prognostic biomarkers for cancer patients^12^. lncRNAs can regulate gene expression via multiple ways, such as chromatin modification, transcription, and posttranscriptional regulation^13^. Emerging studies indicated that lncRNAs were able to function as critical elements of the competing endogenous RNA (ceRNA) network via binding miRNAs to neutralize their suppressing effects on target genes^14^. Recently, numerous lncRNAs have been proved to indirectly regulate gene expression by functioning as ceRNAs in NSCLC progression^15^.

LncRNA titin-antisense RNA1 (TTN-AS1) is transcribed on the antisense strand of TTN and has partial sequence complementarity with TTN. TTN-AS1 has been identified to be an oncogene in numerous cancers, such as hepatocellular carcinoma, cervical cancer, papillary thyroid cancer, and gastric cancer^16–20^. Importantly, TTN-AS1 exerted regulating effects via functioning as a ceRNA to sponge different miRNAs^16–20^. For instance, TTN-AS1 facilitated the proliferation and metastasis ability of esophageal squamous cell carcinoma by the modulating miR-133b/FSCN1 axis^16^. To the best of our knowledge, the expression and significance of TTN-AS1 in LUAD has not been investigated so far. In the present study, we first identified an upregulated expression of TTN-AS1 in LUAD by analyzing the data in The Cancer Genome Atlas (TCGA) database. Then, we detected the expression of TTN-AS1 in LUAD tissues, and found upregulated expression of TTN-AS1 was positively correlated with advanced tumor stage and lymph node metastasis. Knockdown of TTN-AS1 significantly decreased the growth, proliferation, migration, and invasion ability of LUAD cells. In mechanism, we showed that TTN-AS1 could act as a ceRNA to bind to miR-142-5p and indirectly upregulate the expression of cyclin-dependent kinase 5 (CDK5) in LUAD. Herein, TTN-AS1 also exerted promoting effect on epithelial mesenchymal transition (EMT) process of LUAD cells. Taken together, this study unveiled a novel biomarker axis, consisting of TTN-AS1/miR-142-5p/CDK5, which might serve as a novel therapeutic target in treating LUAD.

## Results

### High expression of TTN-AS1 was associated with poor postoperative prognosis of LUAD patients

**Fig. 1:**
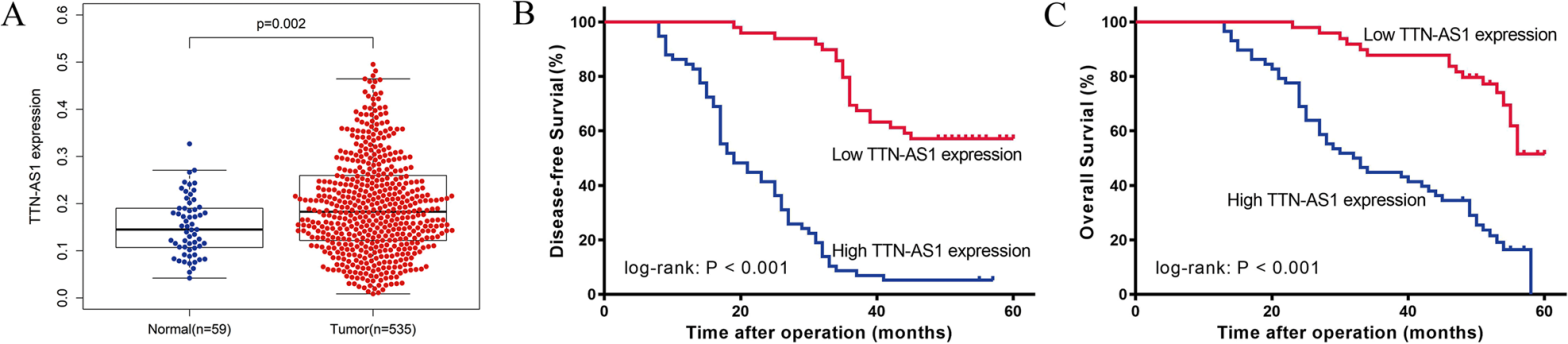
Kaplan–Meier univariate survival analysis of TTN-AS1 expression with a log-rank test for LUAD patients (*n* = 107). **a** Correlation between high TTN-AS1 expression with poor DFS. **b** Correlation between high TTN-AS1 expression with poor OS

To determine the role of TTN-AS1 in LUAD progression, we first explored its expression by analyzing the data from TCGA database. The results demonstrated that TTN-AS1 expression was significantly upregulated in LUAD tissues, compared with normal tissues (Fig. 1a; *P* = 0.003). Then, we determined the expression of TTN-AS1 in 107 LUAD tissues and matched adjacent normal tissues to further confirm the data from TCGA database. Among the total 107 tumor tissues of LUAD patients, 58 cases (54.21%) showed high expression of TTN-AS1, compared with matched adjacent normal tissues. First, we analyzed the association between TTN-AS1 expression and clinical parameters. The results showed that the expression of TTN-AS1 was significantly correlated with the TNM stage and lymph node metastasis while not with gender, age, and invasion range. LUAD patients with higher TNM stage (stage III) and positive lymph node metastasis had significantly higher expression of TTN-AS1 than those with lower TNM stage (stages I and II) and negative lymph node metastasis (Table 1). Furthermore, the role of TTN-AS1 expression in prognosis of LUAD patients was investigated by using Kaplan–Meier analysis and COX proportional hazard regression model. Kaplan–Meier analysis displayed that the high TTN-AS1 expression was correlated with shorter disease-free survival (DFS) and overall survival (OS) (Fig. 1b, c; both *P* < 0.001). Then the COX proportional hazard regression analysis was performed to further assess the predictive role of TTN-AS1 expression in LUAD prognosis. The covariates included in the COX proportional hazard model were gender, sex, invasion range, lymph node metastasis, TNM stage, and TTN-AS1 expression. After stepwise multivariate survival analysis, the TNM stage and TTN-AS1 expression were found to be significantly correlated with the DFS and OS (all *P* < 0.001). In addition, the results showed that the high TNM stage was associated with shorter DFS and OS, with the unadjusted hazard ratio (HR) being 3.130 (95%CI: 1.894–5.171) and 3.702 (95%CI: 2.146–6.389). As to high TTN-AS1 expression, the unadjusted HR being 7.870 (95%CI: 4.537–13.652), and 5.342 (95%CI: 2.937–9.717) for DFS and OS, respectively. The above results indicated that the high expression of TTN-AS1 might be involved with tumorigenesis and regarded as a potential predictor in LUAD patients.

**Table 1:** Associations of TTN-AS1 expression with clinical parameters of LUAD patients

		*n* (%)		*P*
	Total	High expression of TTN-AS1	Low expression of TTN-AS1	
Gender				
Male	49	30 (61.22%)	19 (38.78%)	0.180
Female	58	28 (48.28%)	30 (51.72%)	
Age				
≤60	81	45 (55.56%)	36 (44.44%)	0.393
>60	26	13 (50.00%)	13 (50.00%)	
TNM stage				
I + II	77	36 (46.75%)	41 (53.25%)	0.017
III	30	22 (73.33%)	8 (26.67%)	
Invasion range				
T1 + T2	73	37 (50.68%)	36 (49.32%)	0.306
T3 + T4	34	21 (61.76%)	13 (38.24%)	
Lymph node metastasis				
Negative	38	15 (39.47%)	23 (60.53%)	0.027
Positive	69	43 (62.32%)	26 (37.68%)	

### Knockdown of TTN-AS1 could inhibit growth, invasion, and migration ability of LUAD cells

**Fig. 2:**
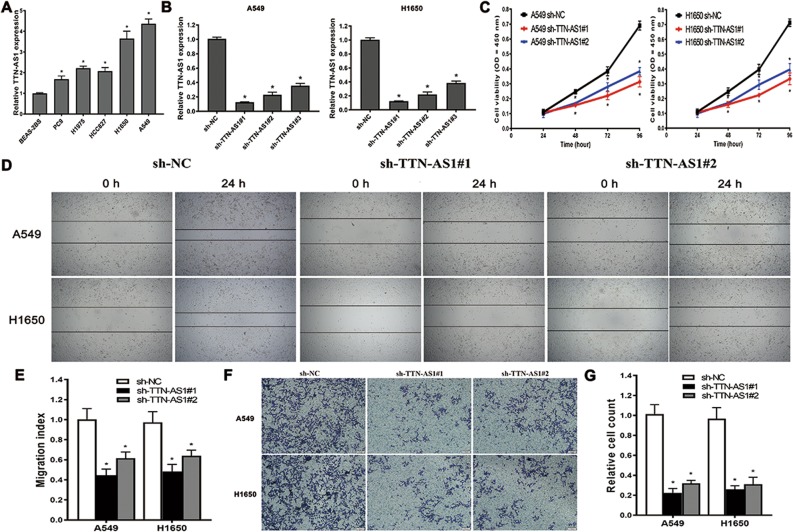
The expression and functions of TTN-AS1 in A549 and H1650 cells. **a** The expression of TTN-AS1 in LUAD and BEAS-2B cells detected by qRT-PCR. **b** The efficiency of three different shRNA sequences in decreasing TTN-AS1 expression in A549 and H1650 cells. **c** The effect of TTN-AS1 knockdown on cell growth ability of A549 and H1650 cells detected by the CCK-8 assay. **d**, **e** The effect of TTN-AS1 knockdown on cell migration ability detected by the wound-healing experiment after 24 h transfection. **f**, **g** The effect of TTN-AS1 knockdown on cell invasion ability detected by the transwell assay after 24 h transfection. *P < 0.05

Aiming at realizing the function of TTN-AS1 in LUAD, we first detected the expression of TTN-AS1 in LUAD cell lines with qRT-PCR. All LUAD cell lines used in this study showed higher expression of TTN-AS1 than the control human bronchial epithelial cell line BEAS-2B (Fig. 2a). Among these LUAD cell lines, the A549 and H1650 cells showed the highest expression of TTN-AS1, so these two cell lines were chosen for next experiments. Then, the expression of TTN-AS1 in A549 and H1650 cells was knocked down with Short hairpin RNA (shRNA) to analyze its biological functions. Among the three shRNA sequences designed for silencing TTN-AS1, the shRNA#1 and shRNA#2 showed efficient inhibiting effect on TTN-AS1 expression in both A549 and H1650 cells (Fig. 2b). Thus, we knocked down TTN-AS1 expression by using shRNA#1 and shRNA#2, and assessed the tumor cell migration and invasion abilities with wound-healing assays, as well as transwell migration and invasion assays. The results demonstrated that TTN-AS1 knockdown with sh-TTN-AS1#1 and sh-TTN-AS1#2 both significantly suppressed the cell growth of both A549 and H1650 cells (Fig. 2c). In A549 cells, the sh-TTN-AS1#1 and sh-TTN-AS1#2 could significantly downregulate the migration index, compared with sh-NC group (0.445 ± 0.060 vs. 1.003 ± 0.107, *P* = 0.001; 0.617 ± 0.061 vs. 1.003 ± 0.107, *P* = 0.006, respectively, Fig. 2d, e). As for the H1650 cells, sh-TTN-AS1#1 and sh-TTN-AS1#2 also significantly downregulated the migration index, compared with the sh-NC group (0.483 ± 0.070 vs. 0.973 ± 0.107, *P* = 0.003; 0.640 ± 0.056 vs. 0.973 ± 0.107, *P* = 0.009, respectively, Fig. 2d, e). Meanwhile, the sh-TTN-AS1#1 and sh-TTN-AS1#2 could significantly downregulate the invasion cell count of A549 cells, compared with sh-NC group (0.223 ± 0.045 vs. 1.013 ± 0.094, *P* < 0.001; 0.320 ± 0.030 vs. 1.003 ± 0.107, *P* < 0.001, respectively, Fig. 2f, g). Likewise, in H1650 cells, sh-TTN-AS1#1 and sh-TTN-AS1#2 also significantly downregulated the migration index when compared with the sh-NC group (0.260 ± 0.036 vs. 0.967 ± 0.112, *P* < 0.001; 0.310 ± 0.070 vs. 0.973 ± 0.107, *P* < 0.001, respectively, Fig. 2f, g). Taken above, TTN-AS1 might have a potential to act as an oncogene in progression of LUAD.

### TTN-AS1 bound to miR-142-5p and decreased its expression in LUAD

**Fig. 3:**
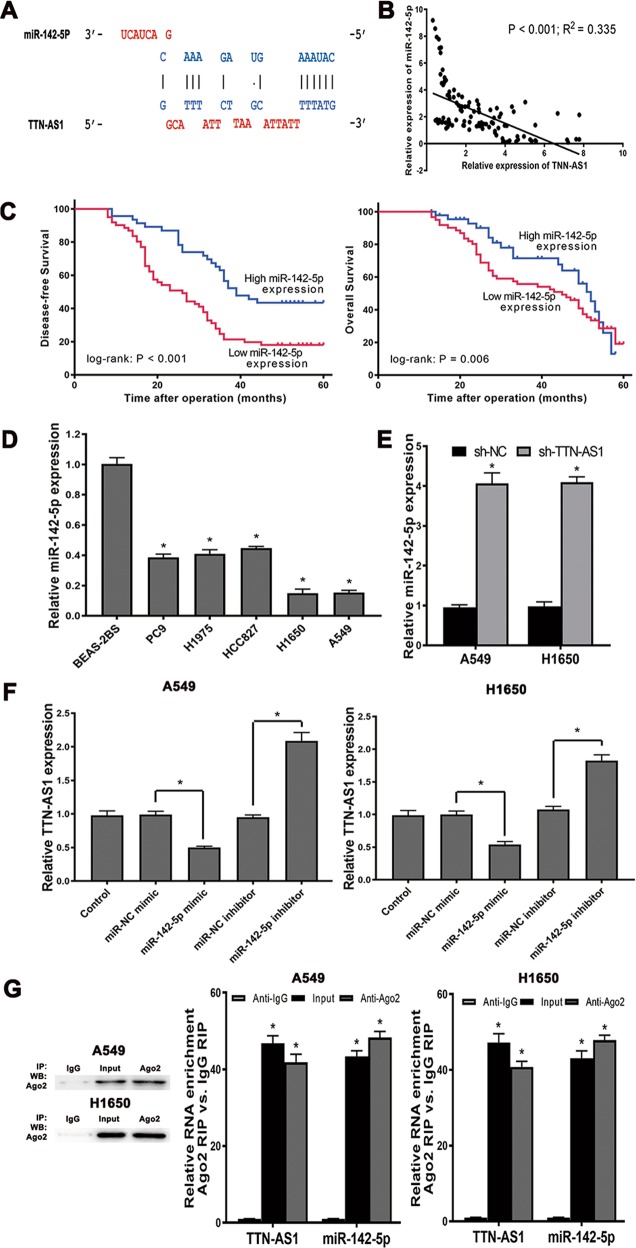
The association between TTN-AS1 and miR-142-5p in LUAD tissues and cell lines. **a** The potential miR-142-5p target sites in TTN-AS1 transcript. **b** The expression correlation between TTN-AS1 and miR-142-5p in LUAD tissues. **c** Example I showing correlation between high TTN-AS1 expression with poor postoperative DFS, and example II showing correlation between high TTN-AS1 expression with poor postoperative OS. **d** The relative expression of miR-142-5p in LUAD cell lines. **e** The effect of TTN-AS1 knockdown on the expression of miR-142-5p. **f** The effect of miR-142-5p mimic and inhibitor on the expression of TTN-AS1. **g** The expression of TTN-AS1 and miR-142-5p in the Ago2-containing beads, compared with the beads harboring control IgG. *P < 0.05

Since many lncRNA can exert various functions in cancers via binding to miRNAs, we then tried to explore the potential miRNAs which might be bound with TTN-AS1. Then, the webserver lnCeDB (http://gyanxet-beta.com/lncedb/) and LncBase V2.0 (http://carolina.imis.athena-innovation.gr/diana_tools/web/)^21^ were adopted to predict potential miRNAs which might be sponged by TTN-AS1. As Fig. 3a demonstrated, there was a potential binding site on TTN-AS1 transcript to interact with miR-142-5p, which provided some evidence for the possible role of TTN-AS1 as a miRNA sponge. Then we assessed the expression of miR-142-5p in LUAD specimens and found that the expression of miR-142-5p and TTN-AS1 were negatively correlated in LUAD specimens (*R*^2^ = 0.335 and *P* < 0.001, Fig. 3b). Kaplan–Meier analysis displayed that the high TTN-AS1 expression was correlated with shorter DFS and OS (*P* < 0.001; *P* = 0.006 and Fig. 3c). For further studying the correlation between miR-142-5p and TTN-AS1, their expressions in LUAD cell lines were detected. As shown in Fig. 3d, low expression of miR-142-5p was observed in all the LUAD cell lines, compared with the BEAS-2B cell. Then, we assayed the miR-142-5p expression in A549 and H1650 cells before and after knockdown of the TTN-AS1. The data demonstrated that after knockdown of TTN-AS1, the expression of miR-142-5p was significantly increased in A549 and H1650 cells (Fig. 3e). Meanwhile, miR-142-5p inhibitor and miR-142-5p mimic could increase and decrease TTN-AS1 expression, respectively (Fig. 3f). Then, aiming at investigating whether both TTN-AS1 and miR-142-5p might be in the RNA-induced silencing complex, RNA immunoprecipitation (RIP) was performed on A549 and H1650 cells. As presented in Fig. 3g, TTN-AS1 and miR-142-5p were substantially enriched (about 42-fold and 48-fold in A549 cells; 41-fold and 48-fold in H1650 cells) in Ago2-containing beads, compared with those harboring control IgG, indicating that endogenous binding might occur between TTN-AS1 and miR-142-5p. In summary, TTN-AS1 could directly bind to miR-142-5p and had the potential to indirectly regulate the expression of the target mRNA of miR-142-5p.

### CDK5 could be downregulated by miR-142-5p in LUAD

**Fig. 4:**
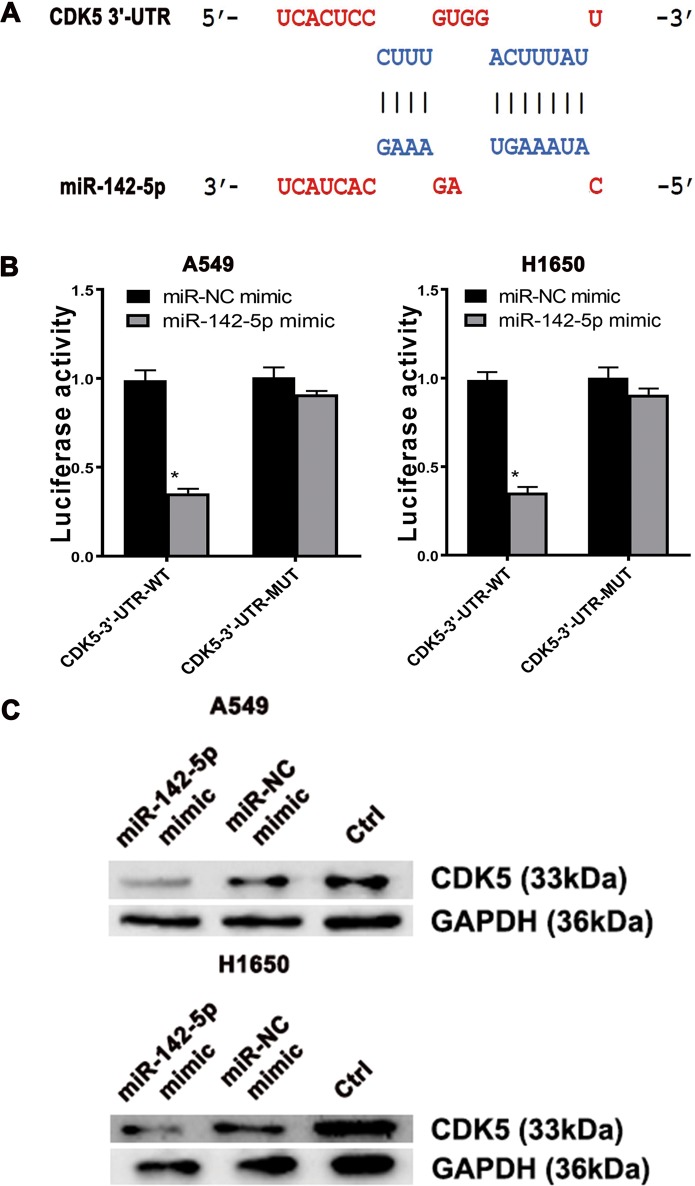
The biological function of TTN-AS1/miR-142-5p/CDK5 axis. **a** The predicted sites in the CDK5 3′-UTR for binding miR-142-5p. **b** The Luciferase reporter assay of A549, and H1650 cells were transfected with the CDK5-3′-UTR-WT or CDK5-3′-UTR-MUT in the miR-142-5p binding sites. Differences were observed when miR-142-5p mimic was added, and miR-NC mimic was used as control. **c** The effect of miR-142-5p mimic on the expression of CDK5 in A549 and H1650 cells

Aiming at further identifying the role of miR-142-5p in LUAD, we used the Targetscan (http://www.targetscan.org/vert_71/) and miRanda (http://www.microrna.org) algorithms to predict potential target of miR-142-5p. CDK5 was predicted to be a target of miR-142-5p in lung. Using the Targetscan and miRanda algorithms, two hypothetic miR-142-5p binding sites were identified in the CDK5 3′-untranslated region (UTR (Fig. 4a). Thus, we performed reporter assays with a luciferase plasmid harboring the 3′-UTR sequence of CDK5 containing the predicted sites for binding miR-142-5p. Furthermore, we built a reporter vector containing a mutation in the miR-142-5p binding sites. These plasmids were transfected into A549 and H1650 cells with miR-142-5p mimic. The results demonstrated that miR-142-5p mimic reduced luciferase activity in A549 and H1650 cells, which were transfected with CDK5-3′-UTR-wild type (WT), but not in those with CDK5-3′-UTR-mutation (MUT) (Fig. 4b). This result revealed that mutation in 3′-UTR of CDK5 could neutralize the repression by miR-142-5p, indicating that miR-142-5p specifically targeted the binding sites in the CDK5 3′-UTR. Meanwhile, CDK5 protein expression was decreased in the presence of miR-142-5p in A549 and H1650 cells, which was consistent with the result of luciferase reporter assay (Fig. 4c).

### The function of TTN-AS1/miR-142-5p/CDK5 axis in LUAD cells

**Fig. 5:**
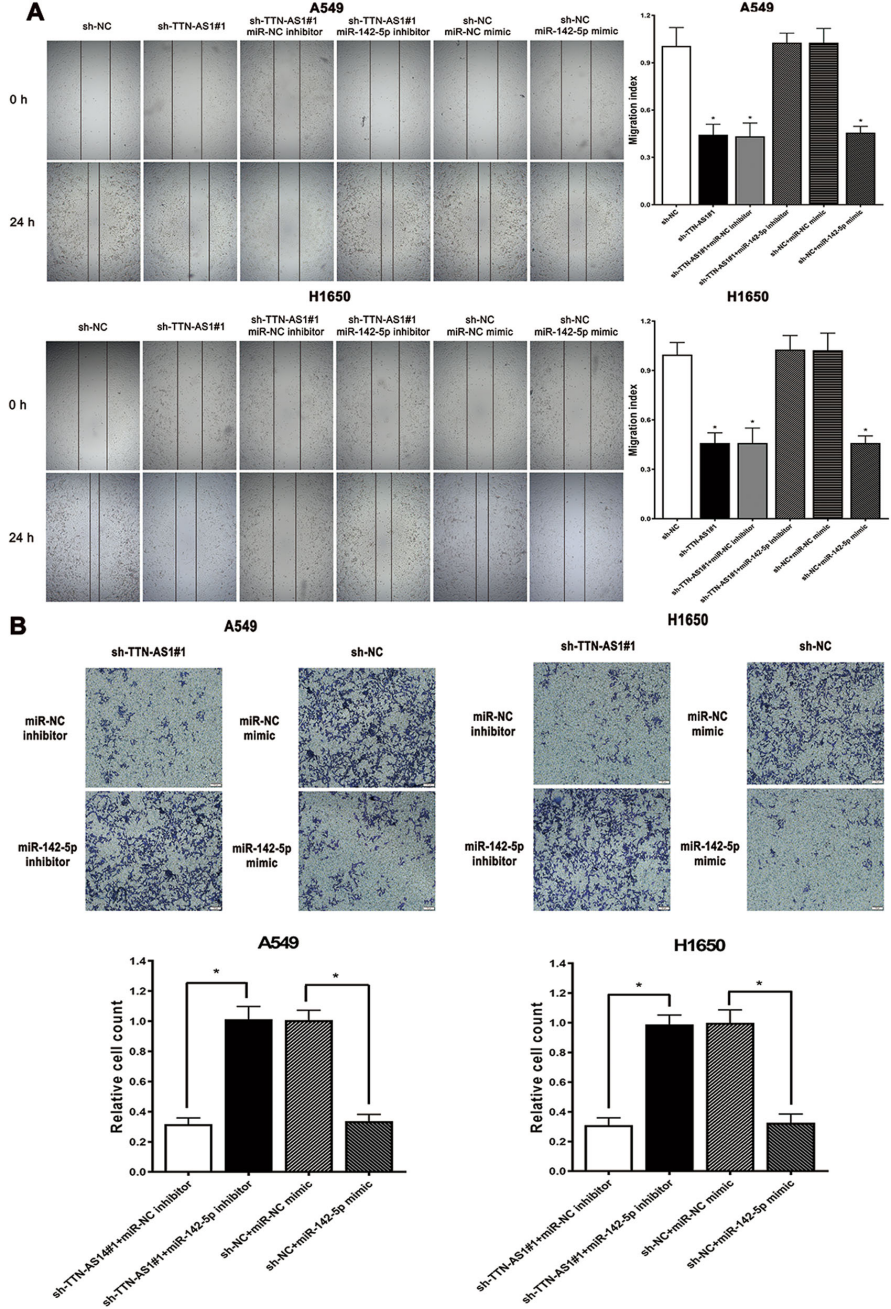
The effects of TTN-AS1 knockdown on migration and invasion ability of A549 and H1650 cells. **a** The effects of TTN-AS1 knockdown on migration ability of A549 and H1650 cells, assayed with wound-healing experiment. **b** The effects of TTN-AS1 knockdown on invasion ability of A549 and H1650 cells, assayed with transwell experiment. miR-142-5p mimic or inhibitor was used for realizing the indirect effect of TTN-AS1 on CDK5. Corresponding miR-NC mimic or inhibitor was used as control. *P < 0.05

To further enhance comprehension of the TTN-AS1/miR-142-5p/CDK5 axis in LUAD cells, we performed the wound-healing and transwell assay to realize its biological functions. The results of wound-healing experiment showed that the migration ability of A549 and H1650 cells could be significantly suppressed by TTN-AS1 knockdown but rescued by inhibition of endogenous miR-142-5p, while miR-142-5p mimic led to a decrease in those cells without TTN-AS1 knockdown (Fig. 5a). Likewise, the data of transwell demonstrated that TTN-AS1 knockdown could inhibit the invasion ability of A549 and H1650 cells, while this inhibition could be rescued by neutralization of endogenous miR-142-5p, and miR-142-5p mimic led to a decrease in those cells without TTN-AS1 knockdown (Fig. 5b). These results demonstrated that TTN-AS1 could enhance the migration and invasion ability of LUAD cells by suppressing the function of miR-142-5p.

**Fig. 6:**
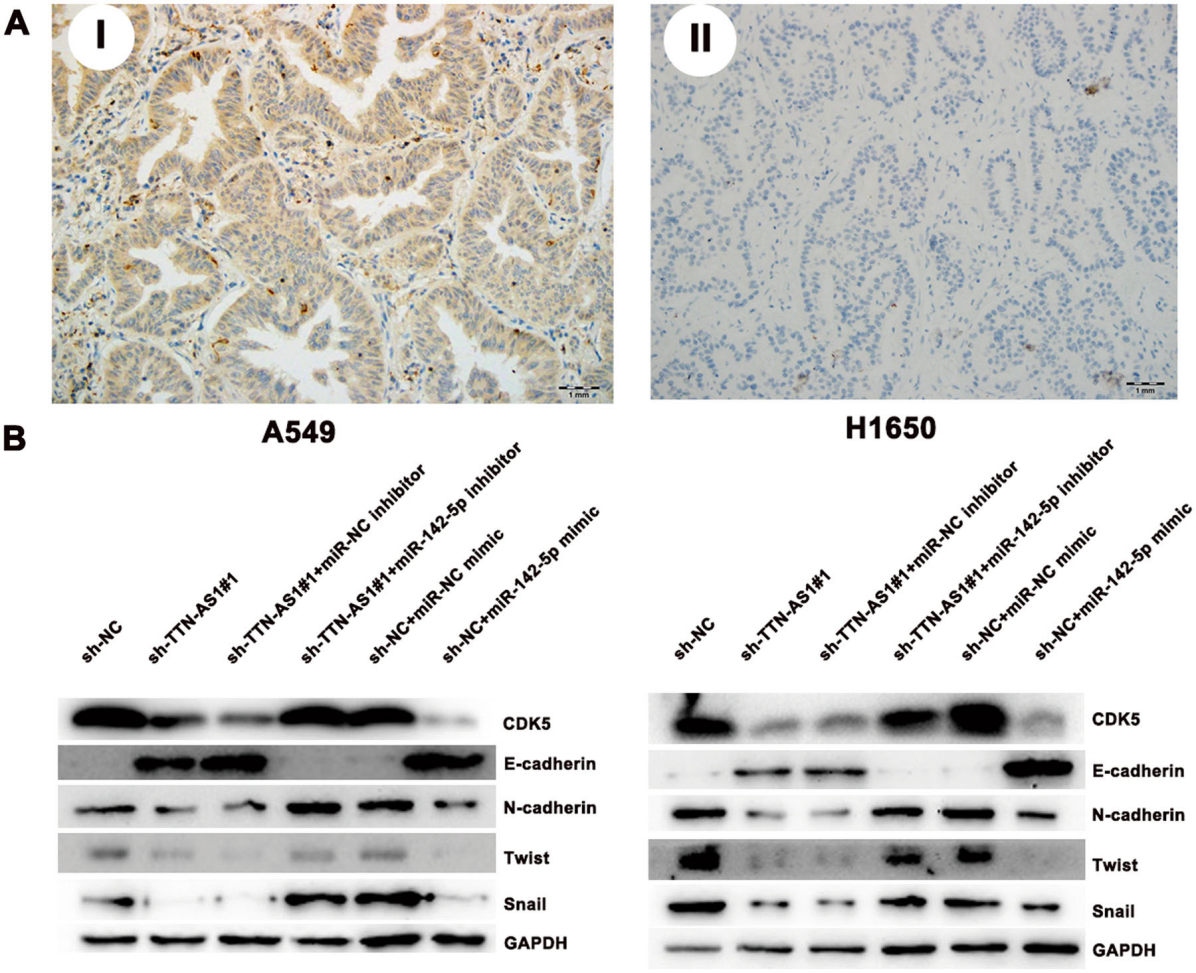
The expression of CDK5 and its correlation between EMT-associated proteins. **a** The representative expression of CDK5. Example I is showing high expression of CDK5, and example II is showing low expression of CDK5. **b** The effect of miR-142-5p inhibitor on the expression of EMT-associated proteins in A549 and H1650 cells with TTN-AS1 knockdown. *P < 0.05

Then, we tried to explore the existence of TTN-AS1/miR-142-5p/CDK5 axis in LUAD, thus, we performed Immunohistochemistry (IHC) to determine the expression of CDK5 in LUAD specimens and analyzed its correlation with TTN-AS1. As Fig. 6a revealed, the staining of CDK5 existed mainly in the cytoplasm of cancer cells. Among the 107 tumor tissues, 61 cases (57.01%) showed positive staining of CDK5. The CDK5 expression was positively correlated with TTN-AS1 and negatively correlated with miR-142-5p expression in LUAD tissues (*P* < 0.001; *P* = 0.001 and Table 2). This result might provide some evidence for the existence of TTN-AS1/miR-142-5p/CDK5 axis in LUAD.

**Table 2:** Association between CDK5 expression with TTN-AS1 and miR-142-5p expression in LUAD tissues

CDK5	TTN-AS1		Spearman’s correlation			miR-142-5p		Spearman’s correlation	
	High	Low	*r*	*P*-value	CDK5	High	Low	*r*	*P*-value
Positive	42	19	0.339	<0.001	Positive	18	43	−0.314	0.001
Negative	16	30			Negative	28	18		

Since CDK5 is reported to be a potential activator of EMT^22^, we then detected the effect of TTN-AS1/miR-142-5p/CDK5 axis on EMT-associated proteins. The results demonstrated that knockdown of TTN-AS1 led to an increase of E-cadherin and decrease of N-cadherin, Twist, Snail and zinc finger E-box binding homeobox (ZEB1) in A549 and H1650 cells, which could be reversed by addition of the miR-142-5p inhibitor (Fig. 6b). Above results indicated that, in LUAD cells, TTN-AS1 could indirectly upregulate the expression of CDK5 to promote the EMT process via inhibiting the function of miR-142-5p. In conclusion, the TTN-AS1/miR-142-5p/CDK5 axis played pivotal roles in the migration, invasion and EMT ability of LUAD cells.

## Discussion

LUAD still remains a malignant disease with unsatisfactory curative effects in the clinical practice. Activation of invasion and metastasis is one of the hallmarks of cancer, which is closely associated with poor prognosis of LUAD paitents^23^. Aberrantly expressed cancer-related genes play pivotal roles in the progression of LUAD. The Encyclopedia of DNA Elements project has confirmed that most of the genome is transcribed as noncoding RNAs, mainly including lncRNAs and microRNAs (miRNAs), etc.^24^ lncRNAs are defined as a class of transcripts longer than 200 nucleotides that lack any detectable open reading frame^5^. Numerous lncRNAs have been confirmed to regulate the expression of target genes through multiple mechanisms^25^. miRNAs are evolutionarily conserved single-stranded RNAs containing about 21–24 nucleotides, which can trigger target mRNA degradation or translation inhibition by targeting its 3′-UTRs^26^. Binding to miRNAs to indirectly regulate the expression of target genes is one of the most frequent function mechanism of lncRNA^15^. Recently, a growing number of studies have elucidated that dysregulated lncRNAs play crucial roles in tumorigenesis of LUAD by participating multiple biological behaviors^27^. For example, the lncRNA linc00673 could bind to miR-150-5p to upregulate ZEB1 and promote EMT in NSCLC^28^. Overall, the interaction between lncRNA and miRNA plays pivotal roles in regulating malignant behaviors of cancers, thus further study on this interaction is beneficial for improving clinical therapeutic efficacy in treating LUAD. However, the function and mechanisms of many lncRNAs have not revealed in LUAD.

In the current study, we examined expression and biological functions of lncRNA TTN-AS1 in LUAD. TTN-AS1, a recently identified oncogene, has been proved to participate the progression of numerous cancers. Chen et al. showed that TTN-AS1 could upregulate E2F3 to promote growth and metastasis ability of cervical cancer via sponging miR-573^18^. Then, Cui et al. verified that TTN-AS1 promoted proliferation, migration, invasion, and EMT in PTC^19^. Lately, Dong et al. found that TTN-AS1 could promote the progression of gastric cancer via functioning as a ceRNA of miR-376b-3p to regulate KLF12^20^. Aiming at realizing the function of TTN-AS1 in LUAD, we first analyzed the expression of TTN-AS1 in LUAD with the help of TCGA database, the results showed that TTN-AS1 was a highly expressed lncRNA in LUAD. Then, to verify the results from TCGA database, we detected the expression of TTN-AS1 in LUAD tissues. Consequently, we found that TTN-AS1 was highly expressed in LUAD tissues comparing with matched adjacent normal tissues. In addition, high expression of TTN-AS1 in tumor tissues was significantly correlated with poor clinical parameters of LUAD patients. Meanwhile, survival analysis revealed that high TTN-AS1 expression was significantly associated with shorter DFS and OS, which suggested that TTN-AS1 might be a potential predictor for poor prognosis of LUAD patients. Aiming at further realizing the biological function of TTN-AS1, we then assayed its expression in LUAD cell lines, and found that A549 and H1650 cells showed the highest expression of LUAD. For realizing the role of TTN-AS1 in LUAD cells, we performed a knockdown of TTN-AS1 with shRNA. As a result, knockdown of TTN-AS1 significantly suppressed the growth, invasion, and migration ability of A549 and H1650 cells. Next, bioinformatics algorithms were used to explore potential binding miRNA of TTN-AS1 to determine its possible functional mechanism. Then, we chose miR-142-5p for our further study. miR-142-5p, identified as a tumor suppressor gene, is proved to be attenuated in various cancers such as osteosarcoma, ovarian cancer, gastric cancer, and NSCLC^29–32^. In the present study, we found that TTN-AS1 shared a complementary binding site with miR-142-5p, thus TTN-AS1 could bind to miR-142-5p and decrease its expression in LUAD cells. In addition, TTN-AS1 and miR-142-5p could be pulled down by anti-Ago2. Taken together, these data indicated that TTN-AS1 could act as a ceRNA to bind to miR-142-5p in LUAD cells.

Aiming to further comprehending the function of TTN-AS1/miR-142-5p axis, we then predicted the potential target of miR-142-5p with bioinformatics algorithms. Then we found that miR-142-5p might target CDK5 by binding to its 3′-UTR region. CDK5, a member of CDK family, was considered to play an important role in numerous cancer-related biological behaviors such as cell proliferation, metastasis, angiogenesis, and immune escape^33–35^. Moreover, Sun et al. also found that CDK5 could induce EMT process in head and neck squamous cell carcinoma^22^. In the present study, we observed that the loss of miR-142-5p was positively correlated with CDK5 expression in LUAD tissues, and further confirmed that miR-142-5p could downregulate CDK5 in LUAD cell lines in vitro. Moreover, we found that the lncRNA TTN-AS1 could bind to miR-142-5p and increase the expression of CDK5 in LUAD cells. Since CDK5 has been reported to be correlated with EMT^22^, thus we next analyzed the correlation between EMT-related proteins and CDK5 in LUAD cells. EMT, a frequent malignant biological behavior, is the process by which epithelial cancer cells lose their cell–cell adhesion, and gain invasive and migratory properties to become mesenchymal stem cells^28^. The EMT program is important for NSCLC to invade their surrounding desmoplastic stroma^36^. E-cadherin is necessary for epithelial cells to maintain cell–cell adhesion, and the loss of E-cadherin is considered to be a fundamental event of EMT^37^. The activation of ZEB1 can downregulate E-cadherin to initiate the EMT process^38^. Then we detected the expression of EMT-related proteins in LUAD cells and found the knockdown of TTN-AS1 led to increased expression of E-cadherin and decreased expressions of N-cadherin, Twist, Snail and ZEB1. Meanwhile, addition of miR-142-5p inhibitor reversed this phenomenon, which confirmed the existence of TTN-AS1/miR-142-5p/CDK5 axis in LUAD. Thus, it could be seen that TTN-AS1 could participate the initiation of EMT through sponging miR-142-5p. As a result, suppression on the invasion and migration ability could be brought by knockdown of TTN-AS1 and rescued by miR-142-5p inhibitor in LUAD cells. Likewise, in the LUAD without knockdown of TTN-AS1, the miR-142-5p mimic could upregulate E-cadherin, and downregulate N-cadherin, Twist, Snail and ZEB1 to inhibit their invasion and migration ability. These results largely expanded the tumor suppressing effect of miR-142-5p and provided more supporting evidence for the ceRNA regulatory network.

In conclusion, To the best of our knowledge, despite of the studies on the role of TTN-AS1 in cancers, this is the first study to analyze its expression and function in LUAD. TTN-AS1 could sponge miR-142-5p to promote migration, invasion, and EMT ability of LUAD cells. High expression of TTN-AS1 was correlated with poor clinical parameters and postoperative prognosis of LUAD patients. Knockdown of TTN-AS1 could restore the suppressing effect of miR-142-5p on CDK5 expression, and led to inhibition of invasion, migration and EMT in LUAD cells. Meanwhile, these phenomena could be rescued by addition of miR-142-5p mimic. The present study revealed that TTN-AS1 might act as a ceRNA to indirectly regulate CDK5 by binding to miR-142-5p. Furthermore, the TTN-AS1/miR-142-5p/CDK5 axis might be used as potential biomarker in predicting prognosis of LUAD patients, and regarded as a novel target for anti-tumor therapies.

## Materials and methods

### Patients and specimens

The specimens of LUAD and matched adjacent normal tissues were collected from 107 patients who underwent surgery at the Fourth Hospital of Hebei Medical University (Shijiazhuang, China) between Jan. 2014 and Jan. 2015. The median patient age at the time of surgery was 51 years (range: 29–73 years). None of the LUAD patients received preoperative treatment including radiotherapy, chemotherapy, or immunotherapy, etc. The clinical stage and histological tumor type were determined according to the UICC Classification of 2009 (seventh edition) and the WHO classification of 2005. Patients’ clinical information were collected and stored in a database, which was updated every 2 months by telephone follow-up. Complete follow-up was updated until death or Jan. 2019. The specimens were collected and treated promptly after surgery. The specimens were frozen and stored at −80 °C for extracting RNA. This research was approved by the ethic committee of the Hebei Medical University and all written informed consents were obtained before surgery.

### Cell culture and treatment

Human LUAD cell line H1650, HCC827, A549, H1975, PC9 and human bronchial epithelial cell line BEAS-2B were obtained from the research center of Peking Union Medical College (Beijing, China). All cells were maintained in RPMI 1640 (Thermo Fisher Scientific, Waltham, MA, USA) containing 5% FBS (Solarbio, Beijing, China), 100 U/ml penicillin and 100 µg/ml phytomycin, at 37 °C in the humidified atmosphere of the 5% CO_2_ incubator.

### The cell proliferation assay

The proliferation of cells was measured by the cell-counting kit-8 (CCK-8) assay according to the manufacturer’s protocol. Briefly, ~2 × 10^3^ cells were plated into 96-well plates. When cells adhered, 10 μl of CCK8 (Solarbio) was added to each well and incubated for 2 h in a humidified incubator containing 5% CO_2_ at 37 °C. The absorbance of each well was detected at a wavelength of 450 nm. Proliferation rates were determined at 0, 24, 48, 72, 96 h after transfection. Experiments were performed in triplicate.

### shRNA, miRNA mimic and inhibitor

Negative control (NC) shRNA and shRNA targeting TTN-AS1 were designed and cloned into lentivirus pCMV-VSV-G by Genepharma (Shanghai, China). The shRNA sequences were listed in Supporting Information Table 1. qRT-PCR analysis was performed to confirm the transfection efficiency. The miR-142-5p and miR-NC mimic and inhibitor were purchased from Thermo Fisher Scientific. For inhibiting or overexpressing miR-142-5p, the cells were transfected with mimic or inhibitor at a final concentration of 25 nmol/l. The cells were planted in 6-well plates for 24 h prior to shRNA or miRNA mimic/inhibitor transfection. The transfections were performed with Lipofectamine 2000 (Invitrogen, Carlsbad, CA, USA) according to the manufacturer’s instruction.

### RNA isolation and quantitative RT-PCR assay

In brief, total RNA was extracted from cells or tissue specimens with TRIzol reagent (Invitrogen) in accordance with the manufacturer’s instructions. Reverse transcription was performed using 1 μg of total RNA with SuperScript RT (Fermentas, Ottawa, Canada). For miRNA detection, the reverse transcription of miRNAs was with special primers (Supporting Information Table 2). Quantitative RT-PCR (qRT-PCR) was conducted with the SYBR Green PCR kit (Takara Bio, Otsu, Japan) on a StepOne Real-Time PCR System (Thermo Fisher Scientific). The comparative threshold cycle (Ct) method was used to calculate the relative expression. The amount of target relative to a calibrator (para-carcinoma tissue expression) is given by 2^−ΔΔCt^ [ΔCt = Ct (target gene) − Ct (internal control gene), ΔΔCt = △Ct (carcinoma) − ΔCt (para-carcinoma)]^39^. Human GAPDH and U6 snRNA were used for TTN-AS1 and miR-142-5p as internal control, respectively.

### Western blot

Cells were lysed in lysis buffer, and protein concentrations were measured with the BCA protein assay kit (Thermo Fisher Scientific). Proteins were separated by 10% SDS-PAGE and transferred electrophoretically onto polyvinylidene difluoride membranes (Millipore, Billerica, MA, USA). The membranes were incubated in PBS containing 5% bovine serum albumin for 2 h at room temperature, followed by overnight incubation at 4 °C with different dilutions of the primary antibodies, including antibodies to CDK5 (EP715Y, Abcam, Cambridge, MA, USA), ZEB1 (ab181451, Abcam), E-cadherin (4A2, Cell Signaling Technology, Boston, MA, USA), N-cadherin (D4R1H, Cell Signaling Technology), Twist (ab50581, Abcam), Snail (C15D3, Cell Signaling Technology) and GAPDH (ab9485, Abcam). The membranes were developed with the Odyssey infrared imaging system according to the manufacturer’s instructions. The levels of protein in each sample were normalized relative to those of GAPDH. Each experiment contained triplicate wells of each sample, and all experiments were repeated at least three times.

### Wound-healing experiments

A total of 5 × 10^5^ A549 or H1650 cells were seeded in 24-well plates. After scraping the cell monolayer with a sterile micropipette tip, the wells were washed three times with serum-free medium. The first image of each scratch was acquired at the time zero. After 24 h, each scratch was examined and captured at the same location, and the healed area was measured.

### The transwell migration and invasion assay

Tumor cell migration assay was performed in a 24-well transwell chamber (Corning, Corning, NY, USA), which contained an 8 μm pore size polycarbonate membrane filter and was precoated with 100 μg Matrigel for invasion assay (Becton-Dickinson, Bedford, USA). Briefly, the cells were seeded in the upper chambers and incubated in 500 μl RPMI 1640 medium without FBS, while 500 μl medium with 10% FBS was placed in the lower chambers. The plates were incubated for 24 h in a 5% CO_2_ humidified incubator at 37 °C. Cells on the upper side of the filters were removed by cotton-tipped swabs, and the filters were washed with PBS. Then the cells on the lower side were fixed in 4% formaldehyde and stained with 1% crystal violet in PBS for 5 min at room temperature. The cells on the lower side of the filters were defined as migration cells and counted at ×200 magnification in 5 random fields of each filter.

### The flow cytometry (FCM) assay

Apoptosis rate was assayed with FCM using the FITC Annexin V Apoptosis Detection Kit I (BD Biosciences, San Jose, CA, USA) according to the manufacturer’s protocol. Briefly, the A549 or H1650 cells were harvested using trypsin without EDTA. After washed twice with precooling PBS, about 5 × 10^5^ cells were resuspended in 100 μl 1× binding buffer, then 5 μl PI and 5 μl FITC Annexin V were added. After incubated for 10 min at room temperature in the dark, 400 μl 1× binding buffer was added to each tube and stained cells were analyzed by FACS Calibur Flow Cytometer (BD Biosciences).

### Dual-luciferase reporter assay

Plasmids containing the firefly luciferase reporter were constructed with CDK5-3′-UTR-WT and CDK5-3′-UTR-MUT. Cells were seeded at 5 × 10^4^ cells/well in 24-well plates and allowed to settle overnight. On the next day, the cells were transfected with recombinant plasmids or an empty plasmid encoding the firefly luciferase reporter with Lipofectamine 2000. Renilla luciferase reporter pRL-CMV (Promega, Madison, WI, USA) was cotransfected into cells as a normalizing transfection control. After 48 h, the reporter luciferase activity was measured with the Dual-luciferase Reporter assay system (Promega) according to the manufacturer’s instructions. All transfection assays were carried out in triplicate.

### RIP assays

RIP assays were performed using the Mana RIP RNA-Binding Protein Immunoprecipitation Kit (Millipore) according to the manufacturer’s instructions. In brief, A549 and H1650 cells were harvested and lysed with RIP lysis buffer containing protease inhibitor cocktail. Then the cell supernatants were incubated with magnetic beads conjugated with antibody against Argonaute 2 (Ago2, C34C6, Cell Signaling Technology) or IgG (AP101, Millipore) for 2 h at 4 °C. Following this, RNase-free DNase I and Proteinase K were consecutively used to remove DNA and protein in the RIP complex. The gained RNA was subjected to qRT-PCR for detecting the enrichment of TTN-AS1 and miR-142-5p.

### The immunohistochemistry assay

IHC analysis was performed as we previously described^40^. The rabbit polyclonal antibody against CDK5 (EP715Y, Abcam) was used for detection. For evaluating expression of CDK5 in LUAD tissues, the staining was visualized and classified based on the percentage of positive cells and the intensity of staining according to the 0–4 semi-quantitative system described in our previous study^40^. The total scores were determined by multiplying the percentage score and intensity score and graded as low for score of 0–4 and high for score of 5–12. Each section was scored independently by two pathologists and a third pathologist determined the final score, if there was any inconsistency.

### Statistical analysis

Statistical analysis was performed by using SPSS statistics software, version 25.0 (SPSS, Chicago, IL, USA). All measurement data were presented as mean ± SD. The association between TTN-AS1 expression and clinical parameters was evaluated using the chi-square test or Fisher’s exact test. Survival analysis was carried out using the log-rank test in association with Kaplan–Meier analysis and Cox proportional hazards model analysis. Student *t* test was used for analyzing the data with equal variance, and Mann–Whitney *U* test was used for those without equal variance. Spearman rank correlation was used to analyze the association between TTN-AS1 with miR-142-5p or between CDK5 with TTN-AS1 and miR-142-5p in LUAD tissue specimens. *P* value < 0.05 was considered as statistically significant, and all *P* values were two-tailed.

## Supplementary information

Supplemental Table S1

Supplemental Table S2
