# Supplementary material for: LncRNA TTN-AS1 promotes migration, invasion, and epithelial mesenchymal transition of lung adenocarcinoma via sponging miR-142-5p to regulate CDK5
Source: Cell Death Dis. 2019 Jul 30;10(8):573. doi: 10.1038/s41419-019-1811-y (PMC6667499; doi:10.1038/s41419-019-1811-y)
Supplement: Supplementary file 1 — Supplemental Table S1 [file 41419_2019_1811_MOESM1_ESM.docx]

Supplemental Table S1. *The sequences of shRNA for TTN-AS1*

|  |  | Sequence |  |
| --- | --- | --- | --- |
| shRNA#1 | Sense | 5’-CACCGCGTTAGTCCACGGAGAATTGCGAACAATTCTCCGTGGACTAACGC-3’ |  |
|  | Anti-sense | 5’-AAAAGCGTTAGTCCACGGAGAATTGTTCGCAATTCTCCGTGGACTAACGC-3’ |  |
| shRNA#2 | Sense | 5’-CACCGCAGATACACAGTGAACATCACGAATGATGTTCACTGTGTATCTGC-3’ |  |
|  | Anti-sense | 5’-AAAAGCAGATACACAGTGAACATCATTCGTGATGTTCACTGTGTATCTGC-3’ |  |
| shRNA#3 | Sense | 5’-CACCGCATCCATGTCCAAACTTACGCGAACGTAAGTTTGGACATGGATGC-3’ |  |
|  | Anti-sense | 5’-AAAAGCATCCATGTCCAAACTTACGTTCGCGTAAGTTTGGACATGGATGC-3’ |  |
|  |  |  |  |
