# Supplementary material for: LncRNA TTN-AS1 promotes migration, invasion, and epithelial mesenchymal transition of lung adenocarcinoma via sponging miR-142-5p to regulate CDK5
Source: Cell Death Dis. 2019 Jul 30;10(8):573. doi: 10.1038/s41419-019-1811-y (PMC6667499; doi:10.1038/s41419-019-1811-y)
Supplement: Supplementary file 2 — Supplemental Table S2 [file 41419_2019_1811_MOESM2_ESM.docx]

Supplemental Table S2. *Primer sequences for qRT-PCR*

| Name | Primer sequence |
| --- | --- |
| miR-142-5p | F: 5’-GAAGATCTCCAGCCACCTGTTTCACA-3’ |
|  | R: 5’-CCGCTCGAGTAGTCCTTCACTTCATG-3’ |
| TTN-AS1 | F: 5’-GCCAGGTAGAGTTGCAGGTT-3’ |
|  | R: 5’-GAAGCTGCTGCGGATGAATG-3’ |
| GAPDH | F: 5’-GTCACCTTCACCGTTCCAGTTTT-3 |
|  | R: 5’-CTTAGTTGCGTTACACCCTTTCTT-3’ |
| U6 | F: 5’-CTCGCTTCGGCAGCACATA-3’ |
|  | R: 5’-AACGCTTCACGAATTTGCGT-3’ |
